# Sustainable Utilization of Coal Gangue in Asphalt Pavement: A Review on Design, Mechanism, and Performance

**DOI:** 10.3390/ma18245666

**Published:** 2025-12-17

**Authors:** Yanshun Jia, Mingyang Lan, Si Peng, Wang Zhang, Chundi Si, Jie Yu, Jiupeng Zhang, Yi Zhang, Zeqi Chen

**Affiliations:** 1 School of Traffic and Transportation, Shijiazhuang Tiedao University, Shijiazhuang 050043, China; jiaysh@stdu.edu.cn (Y.J.); 1202306102@student.stdu.edu.cn (W.Z.); yizhang@stdu.edu.cn (Y.Z.); 2 School of Civil Engineering, Shijiazhuang Tiedao University, Shijiazhuang 050043, China; 1202401106@student.stdu.edu.cn; 3 Taihang Urban and Rural Construction Group Co., Ltd., Shijiazhuang 050222, China; pysloarius@163.com (S.P.); yj11968@126.com (J.Y.); 4 The Key Laboratory of Intelligent Construction and Maintenance of CAAC, Xi’an 710064, China; 5 School of Transportation, Southeast University, Nanjing 211189, China; 230228852@seu.edu.cn

**Keywords:** asphalt pavement, coal gangue, asphalt mixture, mixtures stabilized with inorganic binders, cementitious material, sustainable utilization, review

## Abstract

Coal gangue, a solid waste from coal mining, has long been underutilized while posing environmental and safety risks. This study reviews the current research progress and future prospects of coal gangue as a resource in asphalt pavement. The physical and chemical properties of coal gangue were summarized, and the environmental issues caused by its accumulation were highlighted. The effects of using coal gangue as aggregates or fillers in asphalt mixture were reviewed, along with its activation methods. The research progress on using coal gangue as an aggregate or a cementitious material in mixtures stabilized with inorganic binders was also examined, emphasizing the effects of binder content and coal gangue properties on mechanical and durability performance. The findings indicate that despite its inferior physical properties, coal gangue demonstrates practical feasibility as a pavement material when appropriately incorporated and activated. Proper content enabled coal gangue to meet asphalt mixture or base material requirements, while excessive content reduced low-temperature resistance and caused structural defects. Activated or modified methods can effectively enhance interfacial interaction, high-temperature stability, or structural densification of coal gangue. Recent studies have expressed enthusiasm for innovative activation or modification methods and AI-based performance optimization, while key challenges remain regarding high activation-energy demand, limited aggregate-related research, and an incomplete understanding of interfacial mechanisms.

## 1. Introduction

**Figure 1:**
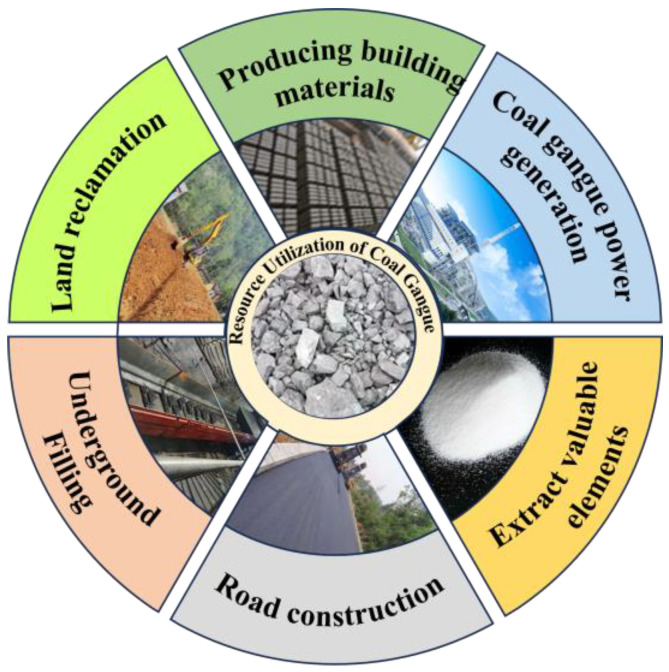
Resource utilization status of coal gangue.

Coal gangue, a byproduct of coal mining and coal washing, is typically disposed of as a solid waste. It is a low-carbon black-gray rock that accompanies coal seams during coal formation and is harder than coal [1]. The utilization of coal gangue can be traced back to the era of infrastructure development in industrialized countries prior to World War II. However, it was not until the 1960s that systematic research in this field began to take shape. In China, the initial use of coal gangue occurred in the mid-20th century, when it was not considered a sustainable resource. The comprehensive utilization and development of coal gangue gradually emerged in the late 1970s [2]. In 2024, the cumulative coal gangue production in China exceeded 4.7 billion tons and continues to grow at a rate of 300–400 million tons annually. Although the comprehensive utilization rate of coal gangue in China has greatly improved in recent years, it remains insufficient compared to the increasing amount of coal gangue produced. It still lags behind that of developed countries, such as the United States and the United Kingdom, where the utilization rate has reached 90% [3,4,5]. The long-term accumulation of coal gangue occupies substantial land resources. Meanwhile, under extended natural weathering, coal gangue can pollute the surrounding atmospheric environment. In addition, under dry and high-temperature conditions, the unburned, carbonaceous organic matter in coal gangue may spontaneously combust due to oxidation, posing serious safety hazards [5]. Therefore, how to effectively dispose of and use a large amount of stockpiled coal gangue has become a key problem to be solved. At present, the comprehensive utilization of coal gangue resources mainly focuses on power generation, the production of building materials, underground filling, gob backfilling, road construction, and land reclamation [5,6], as shown in Figure 1.

In these applications, the road construction consumes a lot of sand and stone resources. The aggregate content within the surface and base materials in asphalt pavements is about 90%, most of which are natural rocks and pebbles [7]. According to relevant statistics by the China Sand and Gravel Association, the national production of sand and gravel in 2024 is about 15.2 billion tons in China, and the overall production has shown a downward trend since 2020 [8]. With the decrease in production, the amount of sand and gravel used in the field of asphalt pavement is also constantly decreasing. Due to its unique porosity and internal composition similar to that of natural aggregates, coal gangue has sufficient potential for application in various structural layers of pavements, and is considered an ideal raw material for road construction. Utilizing coal gangue as a substitute material in road engineering projects can facilitate its rapid utilization, mitigate the escalating scarcity of natural sand, and fulfill, or even enhance, specific pavement performance requirements.

This study aims to systematically review the sustainable utilization of coal gangue as an alternative material in asphalt pavements. Starting from the environmental hazards of stacking coal gangue, the physical and chemical properties of coal gangue and its activation methods are summarized. The effects of coal gangue replacing mineral powder and aggregate on the performance of an asphalt pavement surface and a base layer are explored. The applications of coal gangue in asphalt mixtures and materials stabilized with inorganic binders are summarized, and its potential mechanism and performance trend are analyzed. Finally, the challenges and countermeasures of coal gangue replacing pavement materials are put forward. This review provides theoretical and practical references for guiding the resource utilization of coal gangue and promoting the green and low-carbon development of roads.

## 2. Physical and Chemical Properties of Coal Gangue

The long-term storage of coal gangue not only occupies a large amount of land, but also causes the continuous accumulation of heat inside the coal gangue pile, leading to spontaneous combustion and the release of harmful gases that pollute the surrounding air [9,10]. Coal gangue may release heavy metal elements under weathering and rainwater, polluting the surrounding soil and water. To address the aforementioned risks and promote the resource utilization of coal gangue, it is necessary to start with the basic physical properties and chemical composition to gain a deep understanding of coal gangue.

### 2.1. Physical Properties

**Figure 2:**
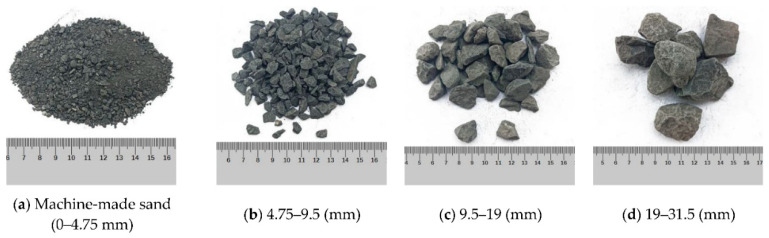
Samples of coal gangue with different particle sizes [12].

**Figure 3:**
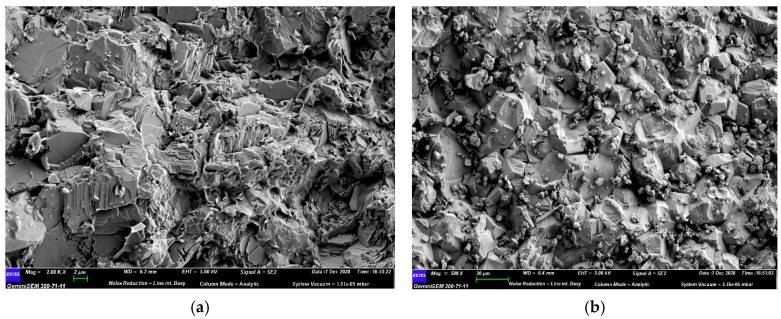
Microscopic morphologies of coal gangue and natural crushed stone [12]: (**a**) coal gangue, (**b**) natural aggregate.

Generally, raw coal gangue in its natural state is black or dark gray, and is often wrapped by slime water and fine coal gangue particles. Spontaneous combustion gangue is formed when the internal temperature of raw coal gangue reaches a certain value due to long-term storage. It usually appears reddish brown or yellowish brown, with a lower carbon content, higher porosity, and exhibits some volcanic ash activity [7,11]. According to the research by Guan et al. [12], the surface of coal gangue particles is predominantly flaky, exhibiting a distinct layered structure with numerous microcracks and pores. The samples of coal gangue with different particle sizes are shown in Figure 2. In contrast, the surface of natural crushed stone particles is closer to a block-like form, generally presenting an irregular polyhedral shape. These particles have higher density, a more compact internal structure, and greater particle integrity. The microscopic morphologies of coal gangue and natural crushed stone are illustrated in Figure 3. Moreover, the crushing value, the content of flat and elongated particles, and the water absorption rate of spontaneous combustion gangue are all higher than those of ordinary coal gangue [13].

Given that particle size distribution is a key factor governing the successful utilization of coal gangue in asphalt pavements, its particle size characteristics are analyzed and compared with those of conventional aggregates. For the particle size of coal gangue, its range is relatively large, and its distribution is uneven. Weathered coal gangue is dominated by coarse aggregate, while the proportions of fine aggregate and filler are relatively low. Related studies have shown that the particle size distributions of coal gangues from different regions are quite different, while the coefficient of uniformity (*C*u) ranges from 2 to 90, and the coefficient of curvature (*C*c) ranges from 0.42 to 9.6, indicating that the problem of poor grading is widespread [14,15,16]. Jiang et al. [17] compared the natural particle compositions of coal gangues from different mining areas and observed an excessive content of coarse particles, while fine particles were insufficient. They also found that particles larger than 5 mm generally accounted for over 60% of the total, with some cases exceeding 80%, whereas particles smaller than 0.1 mm typically comprised less than 5% of the cumulative content. This results in an extremely uneven particle size distribution. Compared with well-graded traditional aggregates (*C*u > 5, 1 < *C*c < 3), coal gangue is difficult to form a continuous and dense structure due to poor gradation. To be specific, the presence of excessive coarse particles leads to a high skeleton porosity, while insufficient fine materials limit the bonding strength of mixtures, exacerbate the risk of mixture segregation, and consequently affect the mechanical stability and durability of the surface/base layer in asphalt pavements. Overall, it can be concluded that coal gangue can replace some natural aggregates, but the impact of gradation on the performance of mixtures must be taken into account. In order to achieve the optimum result, it is essential to optimize the gradation design in specific applications.

Existing studies have indicated that the water absorption ranges from 1.4% to 24.9%, the crushing value ranges from 21.3% to 40.3%, and the content of flat and elongated particles ranges from 13.5% to 33.2% for coal gangue [15,18,19]. These indicators vary from region to region. Compared with natural gravel aggregate, the apparent density of coal gangue aggregate is usually lower [20,21], which is due to the existence of more pores and microcracks. These pores and microcracks also cause the water absorption of coal gangue to be higher than that of natural gravel; therefore, the former needs more water when using them to prepare pavement base materials [18]. In addition, raw coal gangue has a high carbon content, with an average range of 20% to 30% [22]. This high carbon content significantly affects the aggregate’s strength, water absorption, and crushing value. The excessive carbon content is detrimental to the formation of strength for a base layer in pavement materials; this is because, as the carbon content increases, the apparent density, water absorption, and crushing value of the aggregate also increase. Generally, calcination is one of the effective measures for carbon removal, which improves the performance of coal gangue aggregate. However, a previous study has shown that calcination treatment also leads to a significant increase in the water absorption rate of coal gangue [13]. In addition, the crushing value, the content of flat and elongated particles, and the water absorption rate of spontaneous combustion gangue are higher than those of ordinary coal gangue [13], indicating that the material properties of spontaneous combustion gangue are worse than those of ordinary coal gangue in the field of asphalt pavements.

In summary, the surface structure of coal gangue is distinctly layered, with high porosity and poor natural particle gradation. The *C*u and *C*c vary significantly, and the material can be substituted using a volumetric method in base layer applications. The crushing value, content of flat and elongated particles, water absorption rate, and other properties of coal gangue aggregates meet the requirements set out in the relevant specifications for asphalt pavement, but the aforementioned indexes are generally higher. The high carbon content within coal gangue significantly affects its strength, water absorption, and crushing value. As the carbon content increases, its suitability for road applications decreases. To satisfy the performance requirements of coal gangue in various pavement structural layers, pre-treatment measures (i.e., activation), such as grinding and calcination, are frequently necessary to enhance or modify its properties. Among them, calcination can effectively reduce the carbon content and improve the strength of coal gangue, but it increases the water absorption. Therefore, it must be carefully weighed in practical applications.

### 2.2. Chemical Composition

Coal gangue can be categorized based on its primary mineral content into clay rock, sandstone, carbonate, and aluminous rock types. Each category exhibits distinct oxide compositions: clay rock gangue contains SiO_2_ (40–70%) and Al_2_O_3_ (15–30%), sandstone gangue has SiO_2_ content exceeding 70%, aluminous rock gangue is characterized by Al_2_O_3_ content above 40%, and calcareous rock gangue contains CaO exceeding 30% [13]. The diversity of coal resources across different regions is influenced by a variety of factors, including local geological structures, sedimentary environments, and vegetation coverage, which vary across different regions. As posited by several research studies [3,5,23,24], the primary mineral components of coal gangue typically include quartz and kaolinite, while other possible minerals include illite, chlorite, muscovite, feldspar, pyrite, siderite, hematite, calcite, etc. The chemical composition and mineral composition of coal gangue from various countries are shown in Table 1 and Table 2.

The data in Table 1 show that the combined content of SiO_2_, Al_2_O_3_, and Fe_2_O_3_ within coal gangue from China is generally greater than 70%, reflecting its aluminosilicate nature. Table 2 confirms this, as kaolinite (up to 67%) and illite (10–30%) are the major clay minerals, accompanied by moderate amounts of quartz (15–35%). The enrichment of kaolinite enhances the intrinsic pozzolanic reactivity after thermal activation, while quartz contributes to the mechanical strength of the aggregate. Compared with samples from other countries, coal gangue from China typically exhibits higher kaolinite content and lower chlorite abundance, implying superior pozzolanic reactivity. Therefore, regardless of the source of coal gangue, it can be treated as a pozzolanic material [30]. These chemical and mineral features provide a solid basis for its utilization as a supplementary cementitious material or as a partial substitute for natural aggregates, such as limestone and basalt, though region-specific evaluation remains necessary to optimize its performance. It should be noted that the aluminosilicate mineral phase (kaolinite, illite, etc.) is inert in the unactivated, natural coal gangue, and its activity is not stimulated in the natural state, so it cannot play the role of supplementary cementation. It is necessary to take certain activation measures to improve its reactivity and utilization value.

### 2.3. Environmental Risks of Coal Gangue Accumulation

Coal gangue, a solid waste produced during coal mining and washing processes, has long been considered an environmental burden due to its large output and improper disposal. The massive accumulation of coal gangue in open-air dumps not only occupies valuable land resources but also poses multifaceted environmental risks through physical, chemical, and biological interactions with the surrounding environment.

Coal gangue undergoes weathering and oxidation when exposed to atmospheric and hydrological conditions for extended periods. For instance, sulfur- and iron-bearing minerals can be oxidized to produce acidic leachates containing Fe^2+^ and ${\mathrm{SO}}_{4}^{2-}$, which carry various toxic heavy metals such as As, Cd, Pb, and Cr [31,32]. These contaminants may infiltrate surrounding soils and groundwater through rainfall, leading to soil acidification, fertility loss, and the accumulation of heavy metals in agricultural ecosystems [33]. In addition, coal gangue contains radioactive elements such as Hg, Pb, Sr, and U. Under surface geological processes, these radionuclides can be released and dispersed into the surrounding environment, posing threats to both ecological systems and human health [34,35].

In addition, spontaneous combustion is a common phenomenon in gangue heaps with high carbon content. The exothermic oxidation of residual organic matter and pyrite can raise internal temperatures above ignition thresholds, burning and releasing SO_2_, CO, NO_x_, and particulate matter, which lead to regional atmospheric pollution and pose health risks to nearby residents [36,37].

The environmental risks associated with coal gangue accumulation are long-lasting and difficult to mitigate. Heavy metals and acidic components released from gangue weathering persist in soils and aquifers for decades, while the potential for spontaneous combustion remains even after partial reclamation. Consequently, the accumulation of untreated coal gangue represents not only a solid waste management issue but also a persistent ecological hazard that hinders sustainable development in coal-mining regions. Previous studies have demonstrated that the application of coal gangue in asphalt pavements can inhibit the leaching of heavy metals, thereby reducing environmental pollution [38,39]. Moreover, asphalt pavement construction is typically associated with the substantial consumption of natural aggregates. Therefore, the reutilization of coal gangue waste, particularly its incorporation into asphalt mixtures and cement-based materials, has been recognized as an effective approach to mitigate environmental risks while accelerating the consumption of coal gangue stock.

## 3. Application of Coal Gangue in Asphalt Mixtures

From the perspective of structural composition, the macro performance of an asphalt mixture is mainly determined by the structural stability of the aggregate skeleton and the viscoelasticity of asphalt mortar. The stability of the skeleton structure is mainly affected by the physical and mechanical properties and grading of aggregates, while the performance of mortar is mainly affected by the properties of asphalt and filler, the interaction between them, and the filler content [40,41,42,43]. On this basis, coal gangue can replace traditional road materials in terms of coarse aggregate, fine aggregate, and filler.

Ordinary crushed stone aggregates are prepared by crushing stones such as limestone, basalt, and diabase. The coarse and fine aggregates of coal gangue are prepared by crushing and pretreating the coal gangue produced in the process of coal mining and washing. The application of coal gangue instead of natural stone in an asphalt mixture can not only realize the large consumption of coal gangue but also reduce the pressure of large-scale demand for stone in road construction. Unfortunately, due to its poor particle shape, low weathering degree, flaky particles, and low strength, the use of coal gangue to replace coarse and fine aggregates of asphalt mixture has great limitations, and few studies exist. Hu et al. [44] selected coal gangue with a low carbon content, high hardness, and good wear resistance to replace part of the limestone to produce an asphalt mixture. After verification, all properties met the specification requirements, indicating that the use of coal gangue is feasible. Li et al. [45] discussed the feasibility of using coal gangue as a coarse aggregate of an asphalt mixture. The experimental results showed that, although the index of coal gangue is not as good as that of natural aggregate, the performance of the asphalt mixture can still meet the specification requirements when the content of coal gangue is controlled within 30%. In addition, they found that a 6 h treatment with sodium methylsilicate solution yielded the optimal modification effect, significantly reducing water absorption and crushing value while increasing the density of coal gangue.

**Figure 4:**
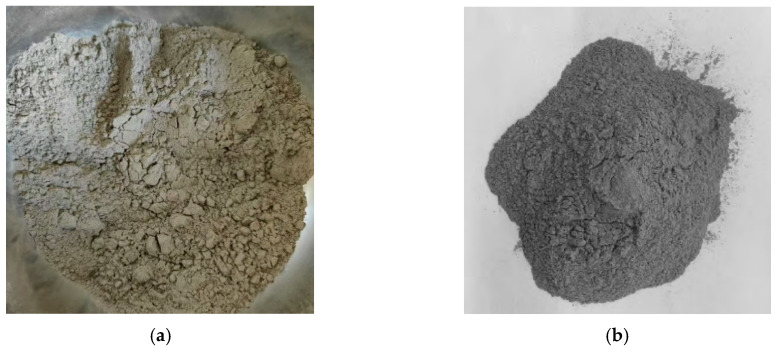
Powdered coal gangue: (**a**) CGP, (**b**) activated CGP [46].

In contrast, recent studies have increasingly focused on material replacement and modification using powdered coal gangue. Powdered coal gangue can replace traditional mineral materials as a new filler, which has two forms: coal gangue powder (CGP) and activated CGP, as illustrated in Figure 4. CGP is made by grinding, and activated CGP is prepared by various activation methods. Activation can modify the surface of filler particles, which is helpful to improve the interaction between asphalt and filler. This section introduces the methods and mechanisms of the activation of coal gangue, analyzes the effects and influencing factors of powdered coal gangue used as a filler from both macro and micro perspectives, and summarizes its advantages, limitations, and possible countermeasures.

### 3.1. Activation Mechanism of Coal Gangue for Asphalt Mixtures

As revealed by the analysis of mineral composition in Section 2, coal gangue has a stable chemical structure and potential active components (e.g., kaolinite). In the composite application of coal gangue with asphalt, the natural activity level of coal gangue is often inadequate to fully meet the requirements for interface bonding and mixture performance. Consequently, coal gangue usually needs to be activated to enhance its applicability. At present, the commonly used activation methods by researchers include mechanical activation, thermal activation, chemical activation, and composite activation. The main goal of these methods is to enhance their bonding ability with asphalt and reduce the mixing temperature of asphalt mixtures to optimize road performance.

Mechanical activation is a conventional pretreatment technique that involves the physicochemical modification of coal gangue through grinding or ball milling. Mechanical grinding has been demonstrated to reduce the particle size of coal gangue, increase the specific surface area, reduce hydrophilicity, and thus enhance adhesive compatibility with asphalt. Zhu et al. [47] investigated the mechanism of mechanical activation in coal gangue and reported that, with prolonged milling time, *C*u first increased and then decreased, while *C*c first decreased and then increased. This indicates that the particles were initially fragmented into irregular fine grains, followed by gradual smoothing of their edges and corners, leading to a more uniform particle size distribution. Furthermore, Fourier transform infrared (FTIR) spectroscopy indicated a progressive depletion of hydroxyl groups during mechanical activation. This finding suggests a reduction in surface hydrophilicity, which can reduce the damage of water to the filler-asphalt interface. Consequently, the filler becomes easier to wrap by asphalt. In addition, mechanical activation is usually a prerequisite for composite activation, as many thermal or chemical activation processes are carried out for coal gangue powder, and mechanical grinding is a necessary step in the preparation of powdered coal gangue.

**Figure 5:**
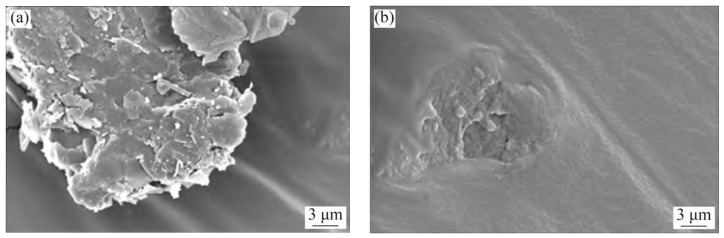
SEM images of asphalt modified by coal gangue [48]: (**a**) CGP, (**b**) activated CGP.

Thermal activation involves the physical and chemical transformation of coal gangue through high-temperature calcination. As observed from scanning electron microscopy (SEM) images by Wen et al. [48], the contact surface between uncalcined coal gangue and asphalt is relatively small, with a distinct interface. This is primarily attributed to its dense layered structure, narrow interlayer spacing, and small specific surface area, leading to poor compatibility with the asphalt matrix. However, after high-temperature calcination, activated mineral components are generated in CGP, expanding its micropores and mesopores [49]. This enables it to adsorb light components in asphalt through capillary action, achieving good compatibility with asphalt, as shown in Figure 5. Jia et al. [50] have shown that CGP, when added to micro-surfacing in the form of a substitute for mineral powder, can improve the stability and durability of micro-surfacing. Among them, CGP activated by a combination of mechanical crushing and high-temperature calcination exhibits better improvement effects. This is also attributed to the coupling treatment, which enhances the activity and surface roughness of CGP, increases its specific surface area and pore structure, and thus strengthens the adsorption effect at the CGP–asphalt interface and the viscosity of the binder. Li et al. [51] have reported the results of road performance tests, indicating that when the calcination temperature is 750 °C, the asphalt mixtures containing CGP exhibit excellent low-temperature crack resistance. Increasing the calcination temperature of CGP fillers is beneficial for improving the creep performance of the corresponding asphalt binders. However, high-temperature calcination increases energy consumption and carbon emissions. The production process of one ton of calcined gangue emits 37–55 kg of CO_2_ [52]. Despite achieving ideal treatment effects, large-scale use is not conducive to resource conservation and environmental protection.

Chemical activation involves a variety of specific methods. There have been studies on modifying CGP using alkaline activators or coupling agents in the application of asphalt mixtures. On the one hand, Wen et al. [48] treated CGP using high-temperature calcination and alkali activation processes, obtaining activated CGP and its modified asphalt. Through SEM, they found that the interface between alkali-activated CGP and asphalt was more blurred. The primary reason for this phenomenon pertains to the formation of aluminosilicate geopolymer by the treatment of coal gangue with wet alkaline dissolution or dry alkaline fusion, which helps increase viscosity and stability of asphalt, and improve compatibility with asphalt. In addition, it was also found that asphalt mixed with alkali-treated CGP had a higher softening point, lower penetration, and a higher rutting factor. This is because activated coal gangue can adsorb more light components of asphalt during the interaction with asphalt, forming spatial constraints that limit the movement of large asphalt molecules. The macroscopic manifestation is an increase in asphalt viscosity and improvement in the high-temperature performance of asphalt. On the other hand, Ding et al. [53] applied a titanate-based coupling agent to modify the surface of CGP, and proved that the treatment effectively enhanced the surface hydrophobicity of the modified coal gangue and improved the mechanical performance and high-temperature stability of the corresponding composite materials. Feng et al. [54] improved the low-temperature cracking resistance of asphalt mastic containing unmodified CGP by introducing the silane coupling agent KH-550. Infrared spectroscopy analysis showed that KH-550 hydrolyzed to silanol groups, which bonded with surface -OH groups on coal gangue and condensed to form a polysiloxane coupling layer on the particles. Experimental results demonstrated that KH-550 modification increased surface roughness and generated additional interstitial pores on the coal gangue particles, thereby enlarging the contact area with asphalt and strengthening interfacial interactions, which further enhanced the pavement-related performance of the asphalt mastic containing activated CGP. In general, chemical activation primarily alters the chemical composition or physical properties of the surface of coal gangue, thereby enhancing the interfacial interaction with asphalt at the microscopic level.

### 3.2. Macroscopic Road Performances of Asphalt Mixtures Containing Powdered Coal Gangue

The tests of replacing part of the filler with CGP have generally achieved ideal results. Zhang et al. [55] analyzed the road performances of asphalt mixtures containing powdered coal gangues with replacement levels of 0%, 20%, 40%, 60%, 80%, and 100%. It was demonstrated that, at a 4% asphalt–stone ratio, the 40% replacement level achieved the highest dynamic stability of 2472 cycles/mm, with an increase in immersion Marshall stability by 1.5 kN, residual stability by 8%, flexural tensile strength by 2.5 MPa, triaxial adhesion by 0.3 MPa, and internal friction angle by 2°. Liu et al. [56] suggested that coal gangue powder can function as a suitable filler in scenarios where high requirements are placed on the dynamic stability, deformation, strength, high-temperature performance, and water stability of rubber asphalt mixtures. Li et al. [57] found that the asphalt mixture mixed with 50% coal gangue, when subjected to microwave heating for 60 s, exhibited not only an excellent crack healing effect, but also a better low-temperature crack resistance, which provides a new direction for the crack repair of hot mix asphalt mixtures. Furthermore, some scholars have considered both the performance of the mixture and its environmental benefits. Wang et al. [58] developed an ecological asphalt mixture that includes coal gangue coarse aggregate and CGP modified with anionic emulsifiers. This mixture has been shown to improve road performance while promoting ecological restoration of the surrounding environment.

The activation of coal gangue has dual effects on road performance. Feng et al. [46] conducted high-temperature performance tests and found that, under the same filler–binder ratio, asphalt mastic incorporating activated CGP exhibited a higher softening point, shear strength, and rutting resistance compared with limestone-based mastic. However, low-temperature tests indicated that replacing mineral filler with an equivalent amount of activated CGP reduces the low-temperature performance of SBS-modified asphalt mastic. Liu et al. [59] concluded that activated coal gangue can improve the conventional properties and high-temperature rheological properties of asphalt. Activated CGP modified asphalt has a significant improvement effect on high-temperature performance and long-term anti-aging performance, but CGP does not improve the low-temperature rheological properties of asphalt.

**Figure 6:**
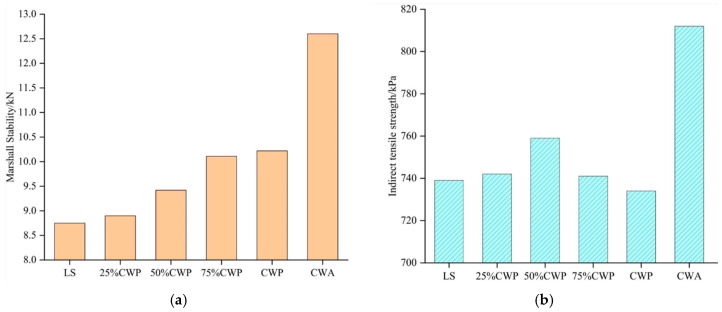
Marshall stability and strength of different asphalt mixtures [7]: (**a**) Marshall stability, (**b**) indirect tensile strength.

In order to intuitively compare the effects of different types of fillers, Feng et al. [60] compared CGP, activated CGP, and limestone powder at five different filler–bitumen ratios. They found that under the same temperature and equal proportions of filler to bitumen, the rutting factor (G*/sin δ, at 64 °C and 70 °C) and Brookfield viscosity (at 135 °C and 175 °C) of asphalt mastic followed the following order: activated CGP > CGP > mineral powder. However, through a softening point test, ductility test (at 15 °C), and bending beam rheological test (at −12 °C and −18 °C), it can be concluded that the order for low-temperature performance was: mineral powder > CGP > activated CGP. Additionally, the Loss on Ignition (LOI) of activated CGP (at 750 °C) is significantly lower than that of CGP, indicating that the raw CGP contains more carbon components, which weaken the interfacial adhesion. High-temperature calcination can effectively remove carbonaceous impurities. Meanwhile, thermal activation induces the dehydroxylation and structural reconstruction of clay minerals in coal gangue (elucidated in greater detail in Section 4.1), producing more SiO_2_ and Al_2_O_3_, as shown in Table 3. Modarres et al. [61] found that compared to mixtures using limestone powder as filler, incorporating powdered coal gangue enhanced asphalt–aggregate adhesion and increased the cohesiveness of the asphalt mastic phase. This enhancement results in higher Marshall stability, indirect tensile strength, and resilient modulus for asphalt mixtures, demonstrating greater toughness. Luo et al. [7] conducted Marshall stability and indirect tensile strength tests for asphalt mixtures with limestone powder, CGP, and activated CGP. The results are compared as shown in Figure 6. In addition, regarding the interaction ability between different fillers and asphalt, they summarized the ranking as follows: CGP > limestone powder > granite powder > fly ash. It can be seen that powdered coal gangue can generally improve the road performance of asphalt mixtures compared to traditional mineral powder, and the use of activated CGP has higher stability and stiffness than other fillers.

### 3.3. Microstructure and Interaction of Asphalt Mixture Containing Powdered Coal Gangue

**Figure 7:**
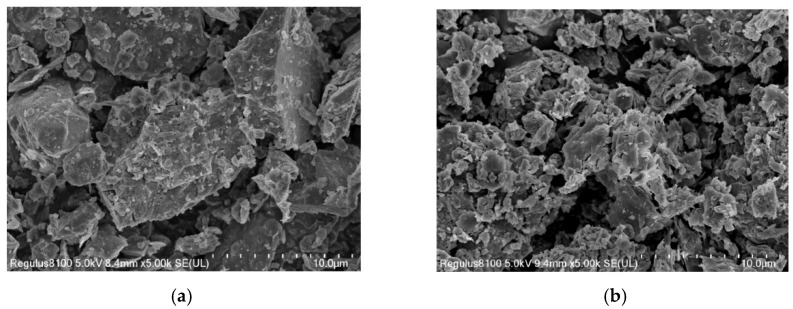
Microscopic images at 5000× magnification [63]: (**a**) mineral powder, (**b**) coal gangue powder.

From the perspective of microstructure, Wang et al. [62] concluded that compared with mineral powder, CGP has a smaller particle size, larger specific surface area, and rougher surface. These characteristics have the capacity to significantly improve the high-temperature performance of asphalt through the surface wetting and interface adsorption of asphalt. Liu et al. [63] reached a similar conclusion. They compared the microstructure of CGP and limestone powder through an SEM test, and the results are shown in Figure 7. Furthermore, the content of active oxides, such as SiO_2_ and Al_2_O_3_, in coal gangue is about 7.8 times that of limestone powder. These physical and chemical properties enable CGP to better adsorb rubberized asphalt. Feng et al. [46] reported that the pores within the activated coal gangue powder can absorb the light components of the SBS-modified asphalt, which in turn impairs its low-temperature performance.

**Figure 8:**
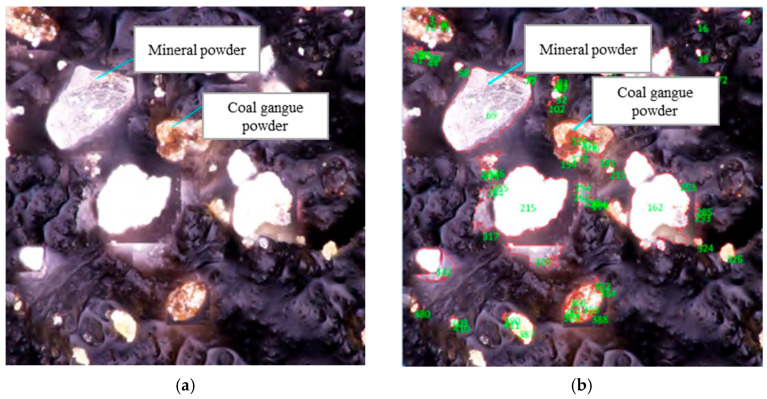
Image-Pro Plus simulation analysis of microstructure of mixture failure [55]: (**a**) mineral powder and CGP after Image-Pro Plus software processing; (**b**) Image-Pro Plus simulation analysis of microstructure during mixture failure.

From the perspective of microscopic interaction, Feng et al. [46] suggested that the rough surface of activated coal gangue powder allows flexible SBS molecular chains to treat the activated particles as “anchoring points,” on which they can attach and entangle. This behavior reinforces the network structure of the SBS-modified asphalt mastic, thereby markedly enhancing its high-temperature stability. Liu et al. [59] indicated that the capillary action of gangue powder allows for part of the asphalt’s light components to infiltrate and mechanically interlock with the pore walls, thereby enhancing the overall performance of the asphalt. Moreover, Wang et al. [64] compared the interaction coefficient *C* value of CGP and asphalt under different powder–binder ratios by using the least square method for parameter fitting. The results indicate that CGP exhibits a higher interaction coefficient with asphalt than limestone powder. Atomic force microscopy was employed to analyze the surface morphology of asphalt mastic. The micromorphology of asphalt mastic containing CGP exhibited approximately twice as many “bee-like structures” as that of asphalt mastic containing limestone powder and formed a denser, intertwined network, which helps to improve cohesion and resistance to permanent deformation. The specific surface area of coal gangue is 2.94 times that of limestone powder, further indicating a stronger interaction with asphalt. A similar conclusion was also reported by Wang et al. [65], and they conducted Fourier transform infrared spectroscopy tests and found that CGP absorbs more light components than limestone powder when in contact with asphalt. This is reflected in an enhanced absorption peak, indicating a stronger interaction between CGP and emulsified asphalt. Additionally, Zhang et al. [55] adopted a new microscopic processing method and used Image-Pro Plus software to process the micro-observation surface of an asphalt mixture with a 40% replacement amount of CGP. The analysis results are shown in Figure 8, in which the green numbers represent the number of mineral powder and activated coal gangue in this microstructure diagram, and the red areas represent the interface and morphology of fracture between mineral powder, CGP, and aggregate when the mixture is damaged after simulation. The results show that the large specific surface area of CGP enhances the bonding of asphalt slurry with coarse and fine aggregates, thereby improving the road performance of asphalt mixtures under an appropriate replacement level.

### 3.4. Limitations of the Application of Powdered Coal Gangue in Asphalt Mixtures

The addition of coal gangue powder (CGP) to asphalt mixtures has both positive and negative effects. While its inherent characteristics of CGP (e.g., large specific surface area, rough surface texture, and dense reticulate structure) improve some aspects of road performance, CGP impairs low-temperature cracking resistance [46,55,61,63,66]. This adverse impact on low-temperature performance mainly stems from two key material-related issues. Firstly, the rough surface and porous structure of CGP result in a high specific surface energy, making it easier for light oil components in asphalt to be adsorbed. This adsorption increases the consistency of the asphalt slurry but compromises its flexibility at low temperatures [55]. Secondly, CGP tends to agglomerate, especially when used in excess. In such cases, asphalt fails to penetrate the internal porous structure of agglomerated CGP, hindering the formation of a dense asphalt mastic matrix and introducing structural defects [66]. These two issues exacerbate stress relaxation behavior and low-temperature cracking. Consistent with these observations, Zhang et al. [55] reported that CGP-modified asphalt mixtures exhibit poorer low-temperature cracking resistance than unmodified mixtures at all replacement levels. Similarly, Feng et al. [60] found that the low-temperature performance of asphalt mortar containing either raw or activated CGP is inferior to mortar with traditional mineral powder.

To reduce the adverse effects of excessive coal gangue content on the low-temperature crack resistance of asphalt mixtures, on the one hand, scholars have studied the appropriate replacement level of CGP. Li et al. [57] used CGP and activated CGP to replace limestone powder at six rates of 0%, 15%, 35%, 50%, 75%, and 100%. The results show that the best fracture-healing property is obtained when the replacement rate of CGP or activated CGP is 50%. This also improves the low-temperature crack resistance of the mixture. On the other hand, some studies have modified the asphalt mixture. Wu et al. [66] found that when the replacement level of CGP was below 50%, the incorporation of polyester fiber promoted effective bridging between coal gangue particles in the asphalt mixture, leading to the formation of a dense grid structure that enhances cracking resistance. They pointed out that the optimum content range of polyester fiber is 0.38–0.42%, with 0.4% being the best. With the decrease in test temperature, the semi-circular specimen of CGP and polyester fiber asphalt mixture specimen changed from plasticity to brittleness. The crack resistance of the specimen increased initially and then declined, with the best performance occurring at −10 °C. Feng et al. [54] modified CGP with a silane coupling agent, thereby rendering the surface of the powder rougher and creating more clearance holes. The resulting increase in contact area with asphalt enhanced interfacial interaction. Li et al. [67] chemically modified coal gangue by a titanate coupling agent and thermal activation. After modification, the tabular structure of coal gangue, induced by crystal phase transformation and chemical grafting, was transformed into a porous structure. This structural change increased the specific surface area, thereby partially reducing fatigue resistance at medium temperatures but simultaneously strengthening the interaction between coal gangue and asphalt.

In summary, by reasonably controlling the content, the performance of an asphalt mixture with coal gangue aggregate can meet the specification requirements, and the performance can be improved by some modification methods. In addition, existing studies indicate that powdered coal gangue, when employed as a filler in asphalt mixtures, exhibits a balance of advantages and drawbacks. At the macroscopic level, it enhances high-temperature stability and adhesion, while at the microscopic level, it strengthens interfacial interactions and structural densification. However, when incorporated at an excessive dosage of CGP, these benefits are offset by adverse effects, such as excessive asphalt absorption and pore-induced defects, leading to low-temperature brittleness and structural weaknesses. Therefore, the engineering value of coal gangue not only depends on its inherent material properties, but also depends on the good performance balance achieved through reasonable dosage control and modification strategy. In addition, the current research is not only limited to improving the road performance of asphalt mixtures through the combination of CGP and modification methods, but also actively explores the self-healing of cracks and environmental remediation. However, the existing research has some limitations. First, it is feasible to replace part of the coarse aggregate of the asphalt mixture with coal gangue, but the existing research lacks in-depth mechanistic analysis and systematic parameter optimization, and the research field of coal gangue as a fine aggregate of asphalt mixture remains largely unexplored. Second, the improvement of low-temperature crack resistance and other properties of an asphalt mixture using coal gangue primarily relies on a single strategy, and more potential modification methods still need to be explored. Finally, at present, the research on the adhesion of asphalt and coal gangue is mostly based on the comparative analysis of the road performance and microinterface with traditional mineral powder, and the adhesion degree of asphalt and coal gangue has not been quantitatively analyzed. In future research, quantitatively analyzing the bonding degree between asphalt and coal gangue (e.g., pull-off test) can be considered, establishing the relationship between the bonding performance of coal gangue asphalt mixture and asphalt, and considering the combination and innovation of various modification methods, so as to further improve the performance of the asphalt mixture.

## 4. Application of Coal Gangue in Materials Stabilized with Inorganic Binders

The selection of pavement base materials needs to take the bearing capacity, stability, economy, and regional resource endowment into account. Materials stabilized with an inorganic binder are widely used in the base course of roads in China. This kind of material uses cement, lime-fly ash, or other materials as the binders, and cements the aggregate or soil by a hydration reaction or pozzolanic reaction to form a semi-rigid/rigid base structure. If the coal gangue can be combined into the base material, it is not only conducive to the reduction in solid waste and resource recycling, but also conducive to reducing the construction cost of the project. The application of coal gangue in inorganic-binder-stabilized materials needs to at least meet or even optimize the use requirements of materials. This section will discuss the research status and application prospects of coal gangue used as road base material from the aspects of its nature, inorganic binder type, inorganic binder content, and coal gangue replacement level. There are two perspectives as follows.

On the one hand, coal gangue can be used as aggregate to replace crushed stone in material stabilized with an inorganic binder [68,69,70]. From the perspective of physical and chemical properties, coal gangue is a kind of hard, gravel-like industrial solid waste with certain strength and skeleton bearing capacity, and its mineral composition is chemically inert, but it has problems such as low density, high water absorption, easy weathering, and high crushing value [14,15,16,17,18,19]. Therefore, researchers regard it as a secondary aggregate with poor performance, but it can still be used. By adding inorganic binders such as cement, lime, and fly ash, the hydration products (C–S–H, C–A–H, etc.) generated by the binders can fill the pores and wrap the coal gangue particles, which can compensate for the lack of strength and durability. From an engineering application perspective, coal gangue can replace natural gravel, reduce material costs, and reduce the pressure on mine resources. When applied to bearing layers, such as base and subbase, attention should be paid to strength, modulus, frost resistance, moisture expansion, etc. And before that, it is necessary to carry out physical modifications, such as screening, crushing, grading adjustment, and pretreatment of the coal gangue. The disadvantages of high variability and weak interface bonding should be key issues in practical applications.

On the other hand, CGP can be used as a cementitious material to partially replace traditional cementitious materials, such as cement and fly ash [70,71,72,73]. The core characteristic of cementitious materials lies in their ability to undergo hydration or pozzolanic reactions to form binding products with cementing capability. As discussed earlier, the main components of coal gangue are similar to those of pozzolanic materials, such as fly ash and metakaolin. Its potential pozzolanic activity—namely, the ability of the Al–Si structure to be destroyed under certain conditions, releasing active oxides that react with Ca(OH)_2_ to generate hydration products—makes it possible for coal gangue to partially or even completely replace traditional cementitious materials. However, due to the stable crystalline structures of natural coal gangue (especially kaolinite, illite, and other components), activation treatments such as thermal or chemical activation are required to enhance its reactivity. These processes, in turn, introduce several challenges, including high energy consumption, reaction instability, and the need for verification of long-term strength and durability. Some researchers have already begun exploring innovative approaches to address these issues. For example, Wu et al. [74] broke the traditional activation technology and proposed a pre-wetted strategy to improve the reactivity and reactivity of CGP in the cementitious system. Li et al. [75] used machine learning to predict the volcanic ash reactivity of coal gangue.

### 4.1. Activation Mechanism of Coal Gangue for Materials Stabilized with Inorganic Binder

Similarly to Section 3.1, in the application scenario of inorganic binder materials, coal gangue also has activation methods such as mechanical activation, thermal activation, and chemical activation, but the activation mechanisms differ. Ca(OH)_2_ is the cement hydration product in the cement-stabilized coal gangue mixture, which can undergo a pozzolanic reaction with the active SiO_2_ and Al_2_O_3_ within coal gangue to generate C–S–H and C–A–S–H, which is beneficial to the overall strength and bonding quality of the mixture [76]. Therefore, in this part, the activation of coal gangue focuses more on improving the pozzolanic reactivity.

Mechanical activation does not generate new chemical bonds [77]; rather, its primary function is to disrupt the original crystalline structure of coal gangue and expose reactive sites. This structural alteration contributes to the refinement of the microstructure of cementitious materials and accelerates the associated chemical reaction processes. Zhu et al. [47] found that under mechanical action, the crystal structure of some mineral phases (such as kaolinite) within coal gangue gradually develops defects and distortions, exhibiting an amorphous state, and this amorphous transition is conducive to reflecting the activation energy. However, mechanical activation has limited effects on altering the chemical structure of coal gangue and requires high energy consumption. Therefore, mechanical activation is often combined with thermal activation or chemical activation.

**Figure 9:**
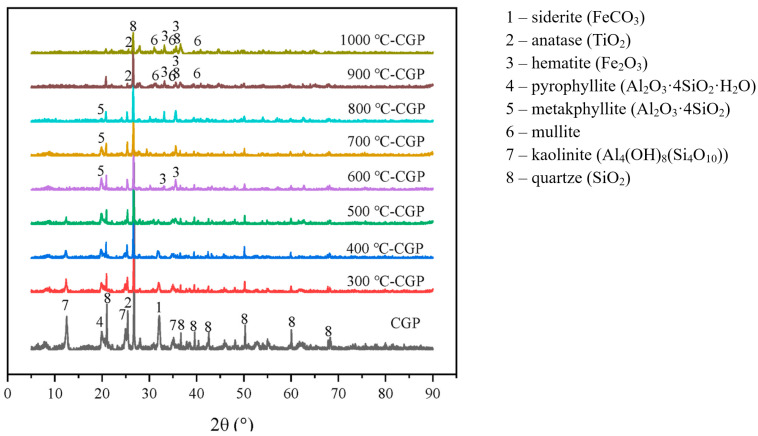
XRD pattern of coal gangue at different calcination temperatures [78].

The thermal activation process primarily targets the kaolinite in coal gangue. Its essence lies in the transformation of the aluminum coordination within Al–O octahedra in kaolinite crystals during calcination, as well as in the depolymerization of Al–O and Si–O tetrahedra. Thermal activation is mainly achieved through high-temperature calcination. Figure 9 [78] shows the X-ray diffraction (XRD) spectra of coal gangue at different calcination temperatures. When the calcination temperature was below 500 °C, the mineral composition of coal gangue was basically unchanged, and the initial dehydroxylation reaction began. When the temperature rose to 600 °C, the diffraction peak of kaolinite disappeared, indicating that the kaolinite part inside the coal gangue gradually transformed into porous, disordered, amorphous metakaolin. At the same time, the stable Si–O and Al–O bonds in the coal gangue began to break, forming a stable silicate structure with pozzolanic characteristics. However, when the temperature was over 900 °C, some of the pyrophyllite decomposed into amorphous SiO_2_ and mullite, and metakaolin began to recrystallize into mullite. This resulted in decreased activity.

In addition to high-temperature calcination, microwave activation is another form of thermal treatment. The principle of microwave activation lies in converting electromagnetic energy into thermal energy, simultaneously heating both the interior and exterior of the material to achieve a uniform temperature distribution. Guan et al. [79] employed microwave irradiation within the temperature range of 600–700 °C to activate coal gangue, promoting the transformation of kaolinite into metakaolinite and thereby enhancing its pozzolanic reactivity. Experimental results demonstrated that incorporating the activated coal gangue into cement mortar increased the 28-day compressive strength by 72.5% compared with the control mortar prepared with untreated coal gangue.

The chemical activation of coal gangue primarily refers to alkali activation. Alkaline reagents such as NaOH, KOH, and Na_2_SiO_3_ can disrupt the aluminosilicate structure of coal gangue, leading to the cleavage of Al–O and Si–O bonds. This process accelerates the dissolution and reaction of coal gangue, thereby promoting the formation of geopolymers and pozzolanic reactions [80]. Geopolymers, also referred to as inorganic polymers, possess a three-dimensional network structure composed of interconnected AlO_4_ and SiO_4_ tetrahedral units. Unlike the hydration reactions of cement or lime, geopolymers are synthesized through the alkali activation of aluminosilicate-rich materials, resulting in a cross-linked framework of AlO_4_ and SiO_4_ tetrahedra [81]. Geopolymers exhibit rapid setting, high thermal stability, recyclability, and low carbon emissions, making them a promising new class of semi-rigid base materials for pavement engineering [82]. However, highly alkaline environments may induce alkaline leaching, posing a challenge to both material performance and environmental safety.

### 4.2. Mechanical Properties of Coal Gangue Stabilized with Different Inorganic Binders

Given its inherent high porosity and large crushing value, coal gangue must be stabilized with an inorganic binder to improve its mechanical properties. Different types of inorganic binders have different impacts on the curing effect of coal gangue. This section summarizes the effects of different types and dosages of the inorganic binders on the mechanical properties of the mixtures containing coal gangue.

**Figure 10:**
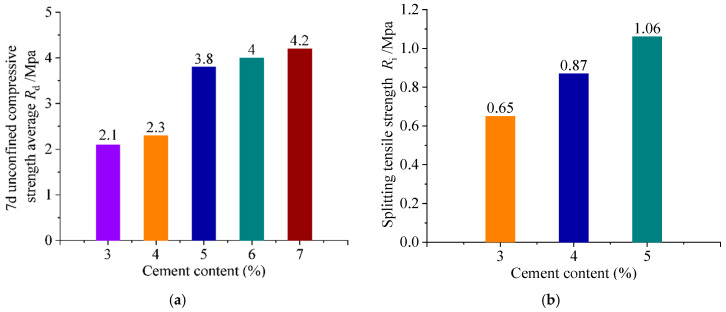
Comparison of unconfined compressive strength and splitting strength of coal gangue at 7 days under different dosages of cement [12]: (**a**) unconfined compressive strength, (**b**) splitting tensile strength.

Firstly, cement has been used as a kind of inorganic binder to stabilize coal gangue. Li et al. [83] used coal gangue to replace ordinary gravel within the range of 2.36–13.2 mm, and used 5.5% cement to stabilize coal gangue. The 7-day unconfined compressive strength of the mixture was greater than 5.0 MPa, and the average compressive resilient modulus of the mixture could reach 1400 MPa, which is within the recommended range of compressive resilient modulus of cement stabilized gravel of 1300–1700 MPa. Wei et al. [84] found through indoor tests that the 7-day unconfined compressive strength of the mixture increases with an increase in cement content. The minimum value recorded was 6.2 MPa when the cement content was 4%, and the maximum value was 9.2 MPa when the cement content was 6%. Guan et al. [12] used 3–7% cement to stabilize coal gangue, and its 7-day unconfined compressive strength was 2.1 MPa–4.2 MPa. As the cement content increased, compressive strength improved, but the rate of increase gradually decreased. The splitting strength of a 90 d cement-stabilized coal gangue mixture increased by 0.22 MPa at 4% cement content and by 0.41 MPa at 5% content compared with 3%. The 7-day unconfined compressive strength and splitting strength of mixtures with different cement contents are shown in Figure 10. Li et al. [76] found that with the curing time from 7 days to 14 days, the strength of coal gangue mixture with 4%, 5%, and 6% cement dosage increased by 36.10%, 30.28%, and 32.42%, respectively. Moreover, the indirect tensile strength of cement-stabilized coal gangue mixtures exhibited a linear increase with their cement content within 90 days. Zhu et al. [85] concluded that the 7-day compressive strength of a cement-stabilized coal gangue base could meet the requirements of medium- and high-grade highways at the dosage of 4–6% cement.

Secondly, lime-fly ash is also regarded as a good inorganic binder. When lime and fly ash are used to stabilize coal gangue, the initial reaction involves the lime reacting with water to form Ca(OH)_2_. The fly ash and active components in the coal gangue undergo a pozzolanic reaction in the Ca(OH)_2_ solution, continuously producing calcium silicate hydrate (C–S–H) and calcium aluminate hydrate (C–A–H) gels. This reaction leverages the pozzolanic components in the coal gangue to enhance the base course strength [86], which is consistent with the findings of Liu [87].

In the latter reaction process, the cement formed a crystalline network structure in the granular gap, which improved the plate property and strength of the coal gangue stabilized by lime-fly ash. Jia et al. [88] observed that when the content of lime and fly ash was below 45%, the comprehensive mechanical properties improved with increasing content, and the proportion of lime and fly ash should be 1:2. Zhou et al. [86] used lime-fly ash to stabilize coal gangue and found that the mechanical strength of the mixture met the standard requirements when 3% cement was added. Meng et al. [89] designed eight groups of coal gangue mixture test proportioning schemes using fly ash, lime, and cement, and conducted water-saturated-unconfined compressive strength tests. The results showed that the strength of materials increased with the fly ash content. The influence of fly ash on strength became more significant with higher cement content, and the beneficial effect of cement on the strength would gradually decrease with the increase in the amount of lime. This indicates that the addition of cementitious materials should be moderate. Zhang [90] applied regression analysis to determine the reasonable dosage range of fly ash and lime, which were 16.1–31.6% and 4–7.8%, respectively. The 60 d compressive resilient modulus and splitting strength of 10 groups of mixtures in the test reached the 180 d strength level of stabilized soil with lime and stabilized soil with lime-fly ash. Moreover, the coal gangue mixtures stabilized by different inorganic binders exhibited substantial differences in mechanical properties, as summarized in Table 4.

In general, there are significant differences in the improvement mechanisms and effects of the different inorganic binders on the mechanical properties of coal gangue mixture. Cement can provide a high and stable strength level, but excessive use will lead to increased brittleness, excessive stiffness, and economic and environmental burdens; the lime-fly ash system can partially stimulate the potential activity of coal gangue, but the strength improvement is limited, and it is difficult to meet the requirements of high-grade roads. Studies have shown that the best solution is not to rely solely on cement or solid-waste cementitious materials, but to achieve a balance between strength and toughness by using fly ash, lime, and other solid waste resources on the basis of 4–6% suitable cement content. In this way, it not only responds to the call for sustainable development of road construction, but also provides a clear boundary and guidance direction for the application of coal gangue base in different grades of roads.

### 4.3. Durability of Coal Gangue Stabilized with Different Inorganic Binders

**Figure 11:**
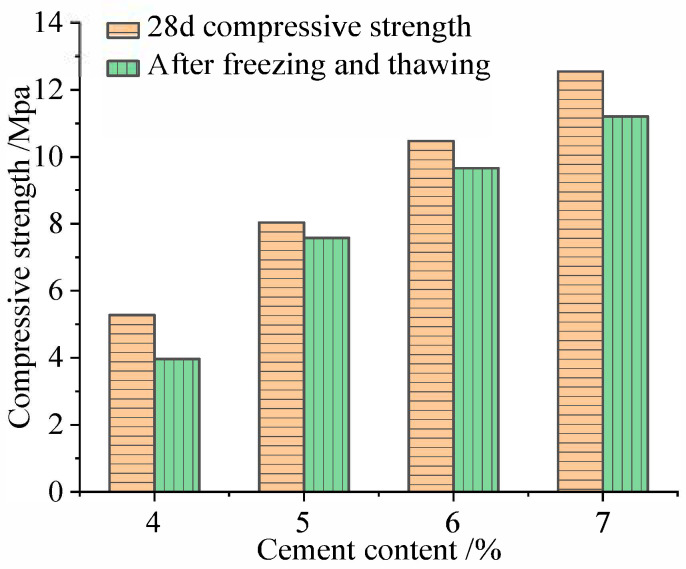
Compressive strength of freeze–thaw cycle mixture of stabilized coal gangue with different dosages of cement [12].

In addition to the mechanical properties, more attention should be paid to the durability of coal gangue stabilized with inorganic binders. The investigation revealed that durability is influenced by the type and content of inorganic binder. On the one hand, some studies [12,76] have demonstrated that after multiple freeze–thaw cycles, the compressive strength loss of cement-stabilized coal gangue exceeded the frost resistance criterion of expressways and Class I highways in heavy-freezing regions (75%), where the mass loss rate is lower than the specification limit (1%). To a certain extent, increasing the cement dosage can reduce the quality loss after the freeze–thaw cycle. Li [76] reported that, after ten freeze–thaw cycles, the mass loss of a coal gangue mixture with 4% cement content was 1.16% lower than that with 3% cement content, while the compressive strength loss decreased by 5.31%. When the cement content increased from 4% to 5%, the mass loss further decreased by 0.87%, and the compressive strength loss was reduced by an additional 3.64%. With the increase in cement dosage, the improvement effect gradually weakened, and the dry shrinkage strain gradually increased. The dry shrinkage strain was the minimum at 4% cement dosage, and the maximum at 6% cement dosage. The results of the freeze–thaw cycle test and shrinkage test of coal gangue mixtures with different cement contents are shown in Figure 11. Furthermore, Zhang et al. [93] prepared eight groups of slag–coal gangue mixtures stabilized with cement and fly ash by substituting coal gangue with slag (<4.75 mm) at different replacement levels and by varying the cement content. The results of the temperature shrinkage test showed that the temperature shrinkage coefficient of the slag–coal gangue mixtures stabilized with cement and fly ash is smaller than that of coal gangue mixtures stabilized with lime-fly ash, which indicated that the mixture can be used as the pavement base of roads in cold regions. The results of the dry shrinkage test showed that slag can inhibit the growth of the dry shrinkage strain of the mixture, while cement can promote an increase in dry shrinkage strain of the mixture. They found that the temperature shrinkage and dry shrinkage coefficients reached their minimum values when the cement content was 5% and the slag replacement level ranged from 50 to 75%.

The durability of coal gangue mixtures can be improved by incorporating certain modifiers. As reported by Zhang et al. [94], incorporating an ion-curing agent into a cement-stabilized coal gangue composite induced charge adsorption, which reduced the thickness of the diffusion layer on the coal gangue surface and increased the availability of free water. This process facilitated cement hydration, promoted the formation of Aft and C–S–H, and decreased the porosity of the mixture, thereby enhancing its compactness. Consequently, the coal gangue mixture demonstrated improved thermal shrinkage resistance, impermeability, scouring resistance, and frost resistance. Many studies [95,96,97] have also incorporated fibers (e.g., polypropylene fiber, basalt fiber) into coal gangue mixtures. These studies have generally resulted in better mechanical and durability properties of the materials.

In summary, a reasonable cement content can alleviate the freeze–thaw damage to a certain extent, but too high a content will amplify the dry shrinkage strain. The study also showed that the incorporation of curing agents or fibers and other modification methods can help to improve frost resistance and water stability. Therefore, the key to the optimization of its durability performance is to control the ratio and combine the modification measures to achieve the coordination and unification of mechanical properties and service durability.

### 4.4. Influence of Characteristics of Coal Gangue on the Performance of Materials Stabilized with Inorganic Binder

After discussing the influence of inorganic binders on material performance, attention should be given to the effects of the intrinsic properties of coal gangue—such as aggregate characteristics, dosage, and gradation—on the overall behavior of the material.

**Figure 12:**
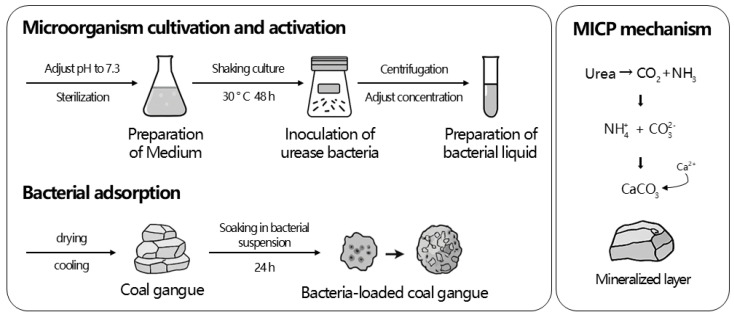
Process and mechanism of MICP-modified coal gangue.

On the one hand, compared with unburned coal gangue, mixtures incorporating spontaneous-combustion coal gangue exhibited superior early-age flexural and compressive strengths. This enhancement can be attributed to its highly porous structure, which promotes asphalt adhesion, and to the presence of reactive mineral phases that accelerate hydration and yield additional cementitious products. Moreover, the physical properties of coal gangue vary significantly across regions, as discussed in Section 2. Coal gangue in different regions has a crushing value of 21.3–44.4%, and the water absorption rate ranged from 1.4% to 24.9% [19]. The crushing value and moisture content of coal gangue have a great influence on the aggregate’s performance. Coal gangue, characterized by a high crushing value, a high proportion of flat and elongated particles, and irregular particle shapes, exhibits a high ellipse ratio and reduced skeleton embedding force and internal friction angle, resulting in mechanical properties inferior to those of natural gravel [98,99,100]. Liu [18] established a prediction model for mechanical properties based on the crushing value of a coal gangue aggregate. It was found that the crushing value was highly correlated with the 7 d and 28 d strength of the mixture, which could be used as one of the reference indexes to measure the mechanical strength. Luo et al. [7] pointed out that the influence of coal gangue aggregate on the strength of the base was in the following order: crushing value > apparent density > silicon–aluminum ratio. It can be seen that the crushing characteristics of coal gangue limit its application in road base course. In addition, high water absorption is also a prominent problem of coal gangue aggregate. Water absorption is second only to crushing value and content of flat and elongated particles in the factors affecting compressive strength [99]. An aggregate with high water absorption will not only increase the optimum water content and reduce the compaction strength, but also lead to greater expansion stress under the action of frost heave, which significantly affects the frost resistance stability. In order to improve its performance, Liu et al. [87] used a selective crushing process to remove low-strength particles, which reduced the crushing value by about 25%. Jia et al. [101] used microbial mineralization (MICP) technology to form CaCO_3_ crystals on the aggregate surface, as shown in Figure 12. The apparent density of coal gangue increased by 14.66%, the water absorption rate decreased by 40.94%, and the crushing value decreased by 7.93%. The strength and crack resistance of the mixture were significantly improved.

**Figure 13:**
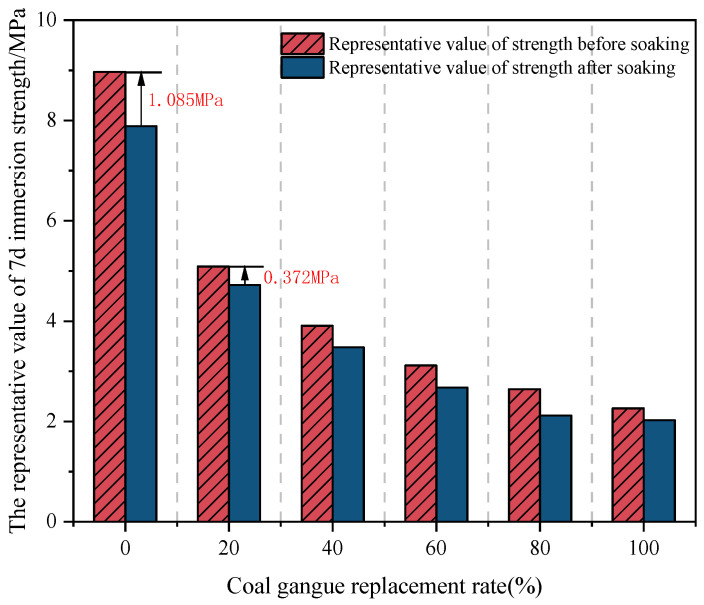
Water stability of cement-stabilized macadam under different coal gangue replacement levels.

On the other hand, the replacement level and particle size selection of coal gangue have a great impact on the mixture. Liu [18] revealed a progressive strength reduction, with mixtures at 20%, 40%, 60%, 80%, and 100% coal gangue replacement levels exhibiting only 58.7%, 43.6%, 37.3%, 31.9%, and 26.2% of the strength of the natural gravel mixture, respectively. The freeze–thaw tests revealed that at a 100% coal gangue replacement ratio, the mass loss rate was 1.92 times that of cement-stabilized natural gravel. When the replacement ratio was below 40%, its influence on the mixture performance was negligible. In the water stability test, the mixture with a lower coal gangue replacement level showed higher immersion strength, due to the adverse effect of coal gangue’s water absorption and expansion behavior on overall water stability, as illustrated in Figure 13. Hence, a high coal gangue replacement ratio should be avoided in practical applications. Meanwhile, it was found that the mass loss rate, compressive strength loss rate, and frost resistance of the specimens with 0–5 mm and 10–20 mm coal gangue were worse than those with 20–26.45 mm and 5–10 mm coal gangue. Similarly, Zhang et al. [102] found that the unconfined compressive strength, splitting strength, and compressive modulus of resilience of the mixture would be low after using coal gangue to replace 0–5 mm and 10–20 mm natural gravel for mix design. Therefore, in practical application, in order to ensure the quality of the project, the use of coal gangue with particle sizes of 0–5 mm and 10–20 mm should be strictly limited to prevent reductions in the compressive and splitting strength of the mixture. The replacement level of coal gangue should be controlled within 45%, and the maximum particle size should be controlled within 13.2–19 mm as far as possible on the basis of meeting the strength [86].

In summary, when coal gangue is used as a road base material, its physical properties significantly affect the road performance of the mixture. Studies have shown that the difference between the crushing value and water absorption rate of coal gangue is mainly due to the internal soft rock, organic matter content, and spontaneous combustion state. The higher porosity and reactivity of spontaneous combustion gangue can enhance the early compressive strength, whereas the improvement of strength in later stages remains limited. The high crushing value and high content of flat and elongated particles for coal gangue weaken the interlocking force of the aggregate skeleton and reduce the internal friction angle of the mixture. High water absorption can increase the optimal moisture content of the mixture, exacerbating the risk of frost heave damage. Selective crushing process or MICP modification can significantly reduce the crushing value and water absorption rate, thereby improving the mechanical properties of coal gangue mixtures. In addition, the substitution rate and particle size selection of coal gangue have a significant impact on strength. When using coal gangue instead of natural gravel for material design, the substitution rate should be minimized, and the use of coal gangue with particle sizes of 0–5 mm and 10–20 mm should be avoided. Coal gangue with a silicon–aluminum ratio of 2–3, high quartz/kaolinite content, and high apparent density should be preferred, and the maximum particle size should be controlled between 13.2 and 19 mm.

## 5. Conclusions and Prospects

### 5.1. Conclusions

As one of the bulk solid wastes, the utilization rate of coal gangue has risen in recent years. The application of coal gangue in asphalt pavement engineering not only effectively solves the environmental pollution and land resource pressure caused by large-scale coal gangue accumulation, but also provides a feasible alternative for natural aggregate. This paper reviews the applications of coal gangue in the materials of asphalt pavement. The main conclusions are as follows:

(1) Coal gangue has high porosity, uneven particle size, high carbon content, and harmful elements. Nevertheless, coal gangue treated by activation, chemical modification, fiber reinforcement, and other means can still meet the requirements of relevant specifications for the material performance of asphalt pavement. In view of its natural defects in physical and chemical properties, coal gangue is destined to be unable to completely replace road materials. In practical application, it is necessary to adopt an appropriate coal gangue replacement scheme based on actual requirements.

(2) Compared with untreated coal gangue, activated coal gangue generally interacts better with asphalt. Therefore, coal gangue typically requires activation to enhance its engineering applicability before being utilized as pavement material. The activity of natural coal gangue is low at room temperature. Composite activation can effectively change the microstructure and chemical composition of coal gangue, and calcination can effectively reduce the carbon content of coal gangue, which has adverse effects on its strength, water absorption, and crushing value. Excessive calcination temperature is deleterious to the performance of coal gangue and increases energy consumption. It is essential to control the calcination temperature within reasonable limits.

(3) By reasonably controlling the content of coal gangue, the performance of an asphalt mixture with coal gangue aggregate can meet specification requirements, and the performance can be improved by some modification methods. In addition, when powdered coal gangue is used as a filler for an asphalt mixture, it shows a balance of advantages and disadvantages. Macroscopically, it enhances the high-temperature stability and adhesion of the material; microscopically, it enhances the interface interaction and structural densification. However, when excessive CGP is added, these benefits will be offset by adverse effects, such as excessive absorption of asphalt and excessive porosity, resulting in low-temperature brittleness and structural defects. When the proportion of CGP replacing traditional mineral powder is about 50%, especially with a polyester fiber dosage of 0.4%, a better performance balance is achieved. Therefore, during the design process of materials for actual road use, particular emphasis should be placed on controlling the dosage and selecting modification strategies.

(4) In the application of pavement base, coal gangue as a substitute for aggregate and cementitious material has shown good application value. Coal gangue shows good cementation potential as the active ingredients in coal gangue can promote the formation of cement-hydration products and improve bonding performance. In addition, the type and content of inorganic cementitious materials have different effects on improving the mechanical properties of coal gangue stabilized by inorganic binder. Cement can provide a higher strength level, but excessive use will lead to increased brittleness, high cost, and environmental burden. Lime-fly ash materials can partially stimulate the potential activity of coal gangue, but the improvement of strength is limited, which makes it difficult to meet the requirements for a high-grade highway. A single inorganic binder is not recommended in engineering practice. The cement content of about 4% is appropriate. On this basis, the comprehensive use of fly ash, lime, and other solid waste resources can achieve better results, so as to achieve the balance of strength and toughness.

(5) The physical properties of coal gangue have a significant impact on the road performance of the mixture. Previous studies show that the difference between the crushing value and water absorption of coal gangue is mainly due to the internal soft rock, organic matter content, spontaneous combustion state, and other factors. The high crushing value and high content of flat and elongated particles for coal gangue weaken the locking force of the aggregate skeleton and reduce the internal friction angle of the mixture. High water absorption can increase the optimal water content of the mixture and aggravate the risk of frost heave damage. Selective crushing process or MICP modification can significantly reduce the crushing value and water absorption of coal gangue, so as to improve the mechanical properties of coal gangue mixture. From the perspective of material selection, when using coal gangue instead of natural gravel for material design, the particle sizes of 0–5 mm and 10–20 mm should be avoided as much as possible, and the maximum particle size should be controlled between 13.2 and 19 mm. Coal gangue with a silicon–aluminum ratio of 2–3, a high content of quartz or kaolinite, and a high apparent density is considered suitable.

### 5.2. Prospects

Despite significant progress, several limitations remain in current studies, along with promising avenues for future research. Firstly, the high-temperature calcination of coal gangue substantially increases energy consumption and carbon emissions, which limits its feasibility for large-scale applications. Future studies should therefore focus more on chemical and composite activation methods, which may achieve comparable or superior activation effects under milder and more sustainable conditions. Secondly, while partial replacement of coarse aggregates in asphalt mixtures with coal gangue has been demonstrated as feasible, most existing investigations remain confined to basic material property tests. Moreover, research on the use of coal gangue as a fine aggregate in asphalt mixtures is virtually unexplored. Hence, future work should aim to overcome the technical barriers that hinder the broader use of coal gangue as an aggregate and promote the development of multifunctional and high-performance composite materials. Thirdly, current studies on the interfacial adhesion between asphalt and coal gangue mainly rely on qualitative or comparative analyses with conventional mineral fillers in terms of macroscopic performance and microstructural interfaces. However, the quantitative evaluation of asphalt–gangue bonding remains limited. Future research should incorporate pull-off and related mechanical tests to quantify the interfacial adhesion strength, while also exploring the synergistic effects of multiple solid wastes to establish a comprehensive understanding of bonding mechanisms in multi-component asphalt mixtures. Fourthly, there remains considerable potential for further development in the gradation adjustment, pretreatment, and optimization of material properties when coal gangue is applied to a pavement base. Finally, with the rapid advancement of artificial intelligence (AI) in pavement engineering, integrating coal gangue-based materials with AI technologies holds vast potential. Data-driven approaches, such as machine learning, can enable intelligent mix design, performance prediction, and multi-field coupling analysis of coal gangue mixtures. Combined with computer vision and intelligent sensing technologies, these methods can facilitate real-time monitoring and degradation prediction, leading to an integrated “design–detection–maintenance” framework. Such integration will advance the intelligent utilization of solid waste resources and promote the development of sustainable and smart transportation infrastructure.

## Figures and Tables

**Table 1:** Chemical components of coal gangue from different habitats.

Areas		Component Content (%)								
		SiO_2_	Al_2_O_3_	Fe_2_O_3_	CaO	MgO	K_2_O	Na_2_O	SO_3_	TiO_2_
China	Shanxi [24]	61.44	26.91	1.05	0.25	0.15	0.42	0.30	-	-
	Hebei [25]	60.75	25.45	4.10	2.01	1.10	2.49	1.33	0.92	1.85
	Jiangxi [26]	58.22	24.77	7.81	1.16	1.50	1.97	1.93	-	-
	Guizhou [27]	40.91	27.78	6.34	7.09	0.78	3.87	-	8.04	3.25
	Anhui [28]	63.07	22.80	6.51	4.32	1.21	-	-	-	-
Spain [29]		43.70	21.35	5.57	0.89	0.77	0.16	0.11	1.02	1.05
Poland [7]		58.95	20.50	6.63	0.35	1.93	3.19	0.54	-	1.05
Italy [7]		43.70	21.40	5.57	0.89	0.77	0.16	0.11	-	1.05
America [23]		47.23	14.61	11.94	4.55	1.68	-	-	-	-

**Table 2:** Mineral compositions of coal gangues from various countries [23].

Minerals	Component Content (%)						
	Belgium	Czech Republic	Germany	Spain	Britain	Russia	China
Illite	80	10–45	41–66	20–60	10–31	5–30	10–30
Kaolinite	12	20–45	4–25	3–30	10–40	1–60	10–67
Chlorite	5	0–15	1–3	0–7	2–7	-	2–11
Quartz	8	10–50	13–27	5–57	15–25	-	15–35
Iron ore	0.5	0–25	0.5–5	-	2–10	0.2–8	2–10
Organic matters	10	0–25	5–10	4–30	5–25	8–40	5–25

**Table 3:** Chemical compositions of CGP, activated CGP, and limestone [38].

Filler	SiO_2_	Al_2_O_3_	Fe_2_O_3_	MgO	CaO	Na_2_O	K_2_O	TiO_2_	*LOI*
CGP	34.80	14.53	3.89	0.87	0.51	0.27	2.39	0.98	40.96
Activated CGP	55.63	23.25	8.09	1.54	2.28	0.59	3.96	1.63	2.01
Limestone	17.59	0.46	0.05	3.64	46.90	0.08	0.10	0.03	29.95

**Table 4:** Mechanical strength and applicable scope of coal gangue mixtures stabilized with different curing agents.

Curing Agent Type		7-Day Unconfined Compressive Strength (MPa)	Applicable Highways	References
Cement	(3%)	3.4	Medium- and high-grade highways	[85]
	(7%)	6.5		
Lime (8%), fly ash (16%)		0.48	Base of low-grade road and subbase of high-grade road	[90]
Red mud, fly ash, desulfurization gypsum	(7 d)	4.18	High-grade highway	[91]
	(90 d)	6.14		
Mineral powder, fly ash, wet calcium carbide slag	(washed coal gangue)	2.18	Medium-grade and below highways	[92]
	(spontaneous combustion of coal gangue)	4.82	High-grade highway	

## Data Availability

No new data were created or analyzed in this study. Data sharing is not applicable to this article.

## Abbreviations

The following abbreviations are used in this manuscript:

CGP	Coal gangue powder
XRD	X-ray diffraction
SEM	Scanning electron microscopy
LOI	Loss on ignition
MICP	Microbially induced calcium carbonate precipitation
AI	Artificial intelligence
